# The Uterus and Its Inflexions

**Published:** 1860-06

**Authors:** 


*The Uterus and its Inflexions.* By PROFESSOR ROKITANSKY.

1. The vaginal portions of the uterus of a multipara, and the connected vaginal roof, is formed out of a duplicature or folding-in of the vagina, in which the lower end of the uterus takes a part. So soon as the uterus has passed into the vagina, the latter surrounds it like a ring, forming an intussusception. In front, the doubling is shorter, and is attached by loose cellular tissue. Hence, the anterior lip is thicker, and the vaginal roof more shallow. After many labors the distinctions become less. On section of the uteri of young persons, it is seen that the vagina, after it has formed the duplicature constituting the roof, is continued into the uterus. The mucous membrane and the layer of the two grow into the corresponding tissues. A second, outer, loose, muscular, longitudinal, fibrous layer of the vagina goes outside, over the duplicature, and spreads over the body of the uterus. In more mature uteri, and those which have been pregnant, there is interposed between the mucous membrane of the cervix and the longitudinal, muscular layer, a richer mass of uterus which ends in a point in the anterior lip of the vaginal portion. At the anterior side of the uterus runs the round ligament, separating into two muscular bands; the upper run together at the fundus, the lower under an angle in the neighborhood of the os uteri internum, thus forming a lozenge-shaped space. At the seat of union of the lower bands, in uteri of this description, there strikes a band of about an inch broad, in the form of a bow, the fasciculi of which fix and enlarge the duplicatures. On the posterior wall of the uterus, the ascending band is continued over the cervix into the vagina; or there proceed, also, from the end of this band, in the neighborhood of the os uteri internum, two strips of the form of a sharp bow, to the vagina. The strong mucous membrane of the cervix, and the thicker connective tissue on the posterior wall, do not terminate at the os internum, but, becoming thinner, go on to the body of the uterus. This forms the support of the mass of the uterus, and the foundation of its upright position, and shares essentially in flexions. Inflexion of the uterus, forward or backward, always falls in the region of the os internum. Flexions of the cervix seldom happen. The stratum of connective tissue is always found less thick, looser, thinner, and even wasted away. Hence, anteflexion is more frequent, and in less degree, as it grows to infraction; retroflection is less frequent, but oftener in extreme degree, and very seldom grows to infraction. Anteflexion, moreever, most commonly appears in the virgin uterus, or at least it is apparently in no relation with labor; retroflexion, on the contrary, hardly ever arises but after repeated labors, or abortions.—(*Allg.*

*Wiener Med. Zeitung*, No. 17, 1859; *Brit. and For. Med.-Chir. Rev.*, January, 1860.)

